# Cloning, Expression, and Purification of Histidine-Tagged *Escherichia coli* Dihydrodipicolinate Reductase

**DOI:** 10.1371/journal.pone.0146525

**Published:** 2016-01-27

**Authors:** Yvonne D. Trigoso, Russell C. Evans, William E. Karsten, Lilian Chooback

**Affiliations:** 1 Department of Chemistry, University of Central Oklahoma, 100 N. University Dr., Edmund, Oklahoma, 73034, United States of America; 2 Department of Chemistry and Biochemistry, University of Oklahoma, 101 Stephenson Parkway, Norman, Oklahoma, 73019, United States of America; La Trobe University, AUSTRALIA

## Abstract

The enzyme dihydrodipicolinate reductase (DHDPR) is a component of the lysine biosynthetic pathway in bacteria and higher plants. DHDPR catalyzes the NAD(P)H dependent reduction of 2,3-dihydrodipicolinate to the cyclic imine L-2,3,4,5,-tetrahydropicolinic acid. The *dapB* gene that encodes dihydrodipicolinate reductase has previously been cloned, but the expression of the enzyme is low and the purification is time consuming. Therefore the *E*. *coli dapB* gene was cloned into the pET16b vector to improve the protein expression and simplify the purification. The *dapB* gene sequence was utilized to design forward and reverse oligonucleotide primers that were used to PCR the gene from *Escherichia coli* genomic DNA. The primers were designed with NdeI or BamHI restriction sites on the 5’and 3’ terminus respectively. The PCR product was sequenced to confirm the identity of *dapB*. The gene was cloned into the expression vector pET16b through NdeI and BamHI restriction endonuclease sites. The resulting plasmid containing *dapB* was transformed into the bacterial strain BL21 (DE3). The transformed cells were utilized to grow and express the histidine-tagged reductase and the protein was purified using Ni-NTA affinity chromatography. SDS/PAGE gel analysis has shown that the protein was 95% pure and has approximate subunit molecular weight of 28 kDa. The protein purification is completed in one day and 3 liters of culture produced approximately 40–50 mgs of protein, an improvement on the previous protein expression and multistep purification.

## Introduction

**Fig 1:**
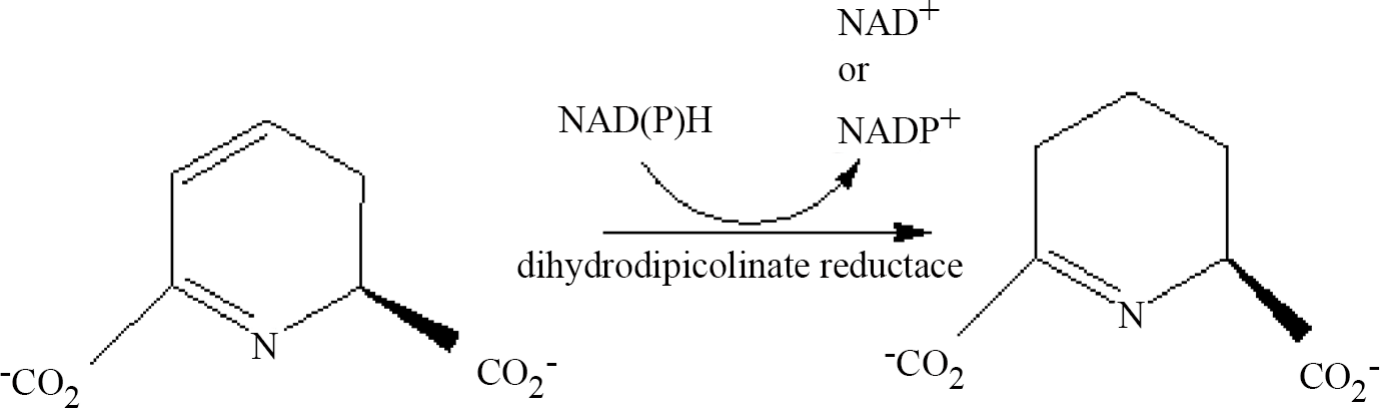
Schematic representation of the reaction catalyzed by dihydrodipicolinate reductase.

Dihydrodipicolinate reductase (DHDPR) was isolated from *E*. *coli* in 1965 by Farkas and Gilvarg [1], since then the enzyme from several different species has been isolated and characterized [2–9]. *E*. *coli* DHDPR gene (*dapB*) encodes a polypeptide of 273 amino acid residues. The resulting protein has a monomeric molecular weight of 28,758 Daltons. The active enzyme is a homotetramer. Dihydrodipicolinate reductase (DHDPR) catalyzes the NAD(P)H dependent reduction of the carbon-carbon double bond of the unsaturated dihydrodipicolinate to form the cyclic imine L-2,3,4,5,- tetrahydropicolinate (Fig 1). The enzyme uses both NADH and NADPH equally well as substrates. The kinetic mechanism of the enzyme is sequential ordered with NAD(P)H binding first followed by dihydrodipicolinate and product release ordered with tetrahydropicolinate released first followed by NAD(P)^+^ [10–15].

Previously, the *dapB* gene from *M*. *tuberculosis* was cloned into the plasmid vector pET22b and transformed into an *E*. *coli* strain in order to characterize and study the structure of M. tuberculosis DHDPR [13]. However, cloning of the *dapB* gene from *E*. *coli* into a histidine-tagged (His-tag) expression vector had not been done before. In this paper we report the cloning of the *E*. *coli dapB* gene into the pET-16b plasmid vector next to the six His-tag sequence and transformed into the *E*. *coli* strain BL21 (DE3), that is ideal for protein expression [16, 17].

## Materials and Methods

Enzymes for DNA cloning and amplification were purchased from Invitrogen (Grand Island, NY). The plasmid pET16b was from Novagen (Madison, WI). Bacterial strain BL21(DE3) was from Invitrogen and manipulated and maintained using standard techniques. The Ni-NTA affinity matrix was purchased from Qiagen (Valencia, CA), or Thermo scientific (Waltham, MA). Buffers and all other chemicals were purchased from SIGMA (St. Louis, MO). Oligonucleotides for the cloning of the reductase gene were synthesized by Invitrogen Laboratory. IPTG was from Gold Biotechnology (St. Louis, MO).

### Cloning of *dapB* into the pET-16b vector

Proximity of the *dapB* gene to the His-tag sequence of pET-16b was kept by utilizing the restriction enzymes NdeI and BamHI. The forward (5’-GAGAATACATATGCATGATGCAAACATCCG-3’) and reverse (5’-GTGGCATGGATCCTTACAAATTATTGAGATC-3’) 30-mer primers for the *dapB* gene were designed accordingly. Chromosomal DNA was obtained from an overnight culture of *E*. *coli* strain JM109 and isolating the genomic DNA using an Invitrogen PureLink Genomic DNA mini kit. The *dapB* gene was obtained by PCR using 1 μg of chromosomal DNA. Thirty cycles of PCR were done using Platinum pfx DNA polymerase. The PCR product was cleaned with the Ultra PCR Clean-Up Kit from Thermo Scientific and subsequently digested by BamHI and NdeI restriction endonuleases. The pET-16b vector was also digested with BamHI/NdeI restriction endonucleases and cleaned using the same kit. The digested plasmid and PCR product were ligated with T4 DNA ligase resulting in the pDHDPR plasmid. The plasmid was transformed into the bacterial strain BL21 (DE3) using standard procedures. The transformed cells were plated on LB plates containing ampicillin (100 μg/ml) and incubated overnight at 37°C. Each plate contained about 50 colonies. The culture plates were stored at 4°C.

Ten colonies were picked from one of the plates and used to inoculate 5 mL of autoclaved LB broth containing 100 μg/ml ampicillin. The cultures were grown overnight at 37°C with shaking at 250 rpm. The pDHDPR plasmids were isolated using a Qiagen DNA Mini-Prep kit. Each plasmid sample was subjected to digestion by BamHI and a double digestion by both BamHI and NdeI. Agarose gel electrophoresis was carried out on the digested products to confirm an 819 bp insert, the expected size of the DHDPR gene. Six out of 10 colonies contained the insert.

### Protein Expression and Purification

*E*. *coli* cell cultures containing the cloned gene were grown overnight at 37°C with shaking at 250 rpm in 5 mL of LB broth containing ampicillin (LB/Amp). The overnight cultures were centrifuged to obtain the cell pellets. Cells were harvested by centrifugation at 4,500 × g. Cell pellets were re-suspended in Ni-NTA lysis buffer (50 mM K_2_HPO_4_, 300 mM NaCl, pH 8.0) and subjected to sonication on ice with a Misonix Ultrasonic Liquid Processors on a 15 second burst cycle (power 10). The cell homogenate was treated with the protease inhibitor phenylmethylsulfonyl fluoride (PMSF), followed by centrifugation to clarify the cell lysate. Supernatants were tested for enzyme activity and the presence of expressed protein was further verified using SDS-PAGE. A stock solution of culture determined to have the highest expression of DHDPR was prepared in 15% glycerol and stored at -80°C. The selected recombinant plasmid was sequenced using the chain termination method at the University of Oklahoma Health Science Center Sequencing Facility.

For protein purification the bacterial stock solution of the recombinant plasmid was cultured overnight at 37°C in 5 mL of LB/Amp broth in a shaker incubator. The culture was divided into equal amounts between three flasks containing 1 L LB/Amp broth and allowed to grow at 37°C to A_600_ = 0.9. The culture was induced with 0.5 mM isopropyl β-D-1-thiogalactopyranoside (IPTG) and growth was continued overnight at 25°C in a shaker incubator. The overnight culture was centrifuged at 4500 × g for 60 minutes to obtain a cell pellet, which was re-suspended in 30 mL of lysis buffer (pH 8.0). The re-suspended cells were lysed by sonication. The sonicated mixture was treated with PMFS and centrifuged for 20 minutes to clarify the cell lysate. The supernatant was added to a Ni-NTA matrix and mixed gently at 4°C for an hour to allow binding of DHDPR. The mixture was batch-cleaned using lysis buffer to remove unbound protein and added to a chromatography column. After the matrix was packed and cleaned with lysis buffer, protein was eluted using elution buffers ranging between 100 mM to 300 mM imidazole in lysis buffer (pH 8.0). One milliliter fractions were collected in microcentrifuge tubes and placed on ice. A Bio-Rad protein assay was done to confirm the presence and relative abundance of protein. The protein peak with reductase activity was separated and placed into a dialysis bag and dialyzed in 50 mM phosphate buffer (pH 8.0) to remove the imidazole. Dialysis was carried out against two one liter volumes of phosphate buffer at 4°C. SDS/PAGE was used to analyze the purity of DHDPR following the purification process. The reductase activity was determined according to the method of Reddy et al. [13]. The reductase was incubated with substrate and NAD(P)H in 50 mM phosphate buffer pH 7.5 and the decrease in absorbance at 340 nm was monitored using a ThermoScientific Evolution 260 Bio UV-Vis spectrophotometer.

## Results and Discussion

**Fig 2:**
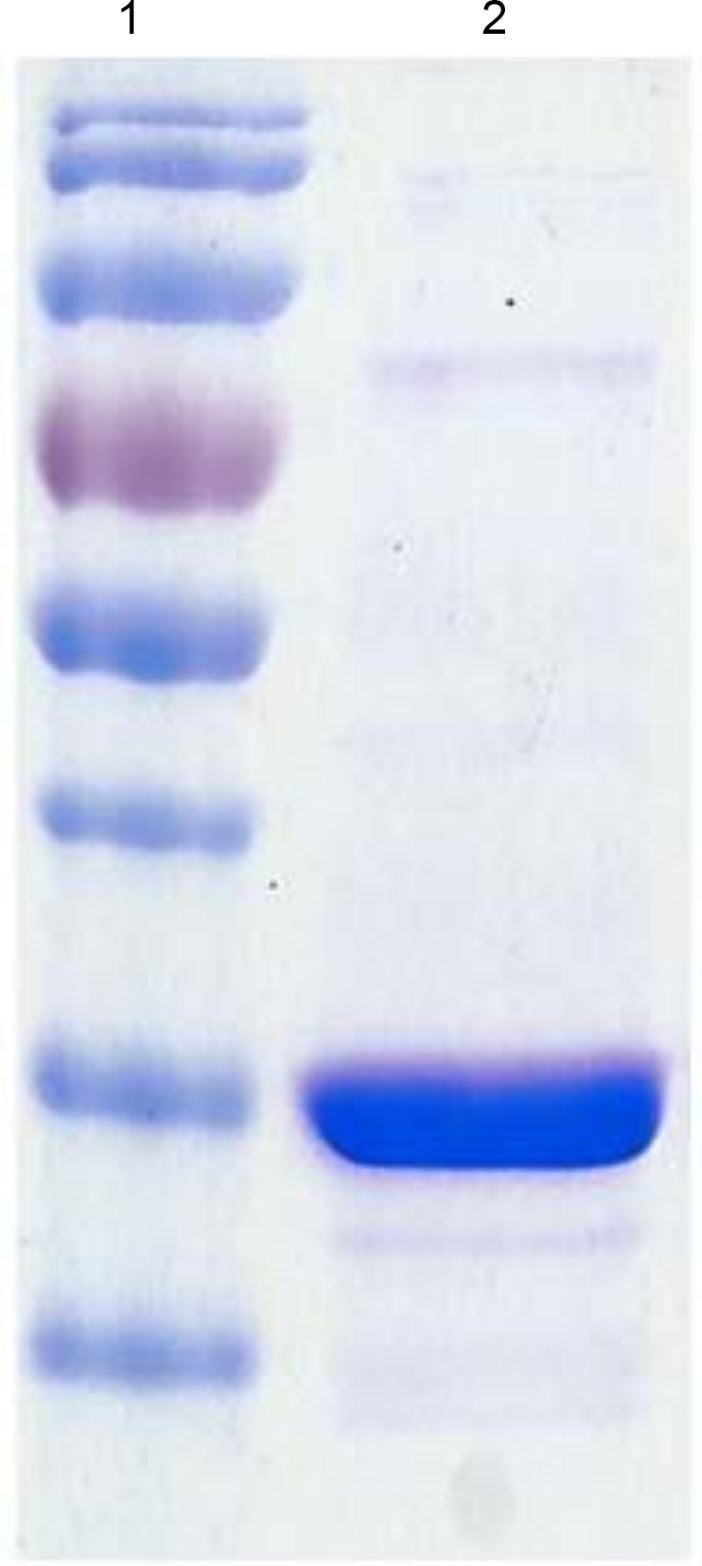
SDS/PAGE analysis of DHDPR. The gel was stained with Coomassie Blue. Lane 1: EZ-Run protein molecular weight marker (170, 130, 95, 72, 56, 43, 34, 26, 17, 11 kDa). Lane 2: purified and dialyzed DHDPR.

DHDPR eluted from the Ni-NTA resin at 200–250 mM imidazole. A sample of the pooled fractions containing DHDPR was analyzed by SDS-PAGE and the results are shown in Fig 2. As shown in the figure, DHDPR (lane 2) migrates to a position in the gel close to the 26 kDa band in the molecular weight markers in lane one and is close to the expected molecular weight of 28 kDa for DHDPR. The protein was estimated to be about 95% pure. Purification of 3 L of culture yielded approximately 40–50 mg of protein.

A DNA fragment containing the *E*. *coli* dihydrodipicolinate reductase gene was successfully isolated and cloned into the histidine-tagged pET-16b expression vector. The 819 bp open-reading frame encoded the 273 amino acid polypeptide with subunit molecular mass of 28 kD. The recombinant histidine-tagged protein was purified from *E*. *coli* in one step using Ni-NTA affinity chromatography. The purification can be completed in one day and represents a significant improvement over the three day purification procedure of the non-histidine-tagged recombinant protein [16].

The kinetic properties of the isolated protein were equivalent to the previously cloned reductase [13, 15]. The specific activity (μmols min^-1^/mg) of the recombinant enzyme reported here is 448 and compares favorably to the published value of 398. In addition, the *K*_m_ for DHDP for the histidine-tagged enzyme is 43 ± 8 μmolar compared to the published value of about 50 μmolar. The histidine-tagged enzyme also uses both NADH and NADPH as substrates similar to what has been reported previously [16].

In our lab the reaction of dihydrodipicolinate synthase (DHDPS) is followed in a coupled assay using DHDPR. In this assay the product of the DHDPS-catalyzed reaction is converted by dihydrodipicolinate reductase to product with concomitant conversion of NADH to NAD^+^. Enzyme activity is monitored spectrophotometrically by measuring the decrease of NADH at 340 nm. The coupled enzyme assay requires relatively high concentrations of DHDPR. The new recombinant produces a high amount of protein which will facilitate kinetic studies of DHDPS.
